# Evaluation of the recombinant proteins RlpB and VacJ as a vaccine for protection against *Glaesserella parasuis* in pigs

**DOI:** 10.1186/s12917-020-02377-5

**Published:** 2020-05-27

**Authors:** Samantha J. Hau, Shi-Lu Luan, Crystal L. Loving, Tracy L. Nicholson, Jinhong Wang, Sarah E. Peters, David Seilly, Lucy A. Weinert, Paul R. Langford, Andrew N. Rycroft, Brendan W. Wren, Duncan J. Maskell, Alexander W. Tucker, Susan L. Brockmeier, on behalf of the BRaDP1T Consortium

**Affiliations:** 1 USDA, ARS, National Animal Disease Center, 1920 Dayton Ave, Ames, IA 50010 USA; 2 Department of Veterinary Medicine, University of Cambridge, Cambridge, UK; 3 Section of Paediatric Infectious Diseases, Department of Infectious Disease, Imperial College London, St. Mary’s Campus, London, UK; 4 The Royal Veterinary College, Hawkshead Campus, Hatfield, UK; 5 Faculty of Infectious & Tropical Diseases, London School of Hygiene & Tropical Medicine, London, UK; 6 Current address: The University of Melbourne, Level 9, Raymond Priestley Building, Melbourne, Victoria 3010 Australia

**Keywords:** Subunit vaccine, Glässer’s disease, *Glaesserella parasuis*

## Abstract

**Background:**

*Glaesserella parasuis*, the causative agent of Glӓsser’s disease, is widespread in swine globally resulting in significant economic losses to the swine industry. Prevention of Glӓsser’s disease in pigs has been plagued with an inability to design broadly protective vaccines, as many bacterin based platforms generate serovar or strain specific immunity. Subunit vaccines are of interest to provide protective immunity to multiple strains of *G. parasuis*. Selected proteins for subunit vaccination should be widespread, highly conserved, and surface exposed.

**Results:**

Two candidate proteins for subunit vaccination (RlpB and VacJ) against *G. parasuis* were identified using random mutagenesis and an in vitro organ culture system. Pigs were vaccinated with recombinant RlpB and VacJ, outer membrane proteins with important contributions to cellular function and viability. Though high antibody titers to the recombinant proteins and increased interferon-γ producing cells were found in subunit vaccinated animals, the pigs were not protected from developing systemic disease.

**Conclusions:**

It appears there may be insufficient RlpB and VacJ exposed on the bacterial surface for antibody to bind, preventing high RlpB and VacJ specific antibody titers from protecting animals from *G. parasuis*. Additionally, this work confirms the importance of utilizing the natural host species when assessing the efficacy of vaccine candidates.

## Background

*Glaesserella parasuis* is a Gram-negative bacterial member of the *Pasteurellaceae* family and the causative agent of Glässer’s disease, which is characterized by a fibrinous polyserositis, meningitis, and arthritis. *G. parasuis* can cause high morbidity and mortality in herds resulting in significant losses to the swine industry annually [1]. There are 15 identified serovars of *G. parasuis*; however, many isolates are untypable [2]. Multiple serovars can circulate within a herd, although it appears some serovars are more capable of causing systemic disease [3, 4].

To prevent *G. parasuis* disease in the swine industry, efforts have focused on developing broadly protective vaccines. Commercially available *G. parasuis* vaccines are predominantly based on a bacterin platform. Bacterins have been shown to provide good homologous protection [5–7]; however, this protection can be serovar or strain specific [7–10], leaving swine susceptible to disease with other serovars or strains in the field. Currently, no available vaccine is able to provide broad cross protection for *G. parasuis*. This may be due in part to the bacterial capsule, which is serovar specific and functions to mask other antigens on the bacterial surface that may contribute to the protective immune response [11, 12]. The importance of a vaccine conferring heterologous protection has led to the pursuit of alternative vaccine platforms that avoid the generation of capsule directed immunity, such as protein and peptide vaccines. Antigens targeted for *G. parasuis* protein and peptide vaccines should be highly conserved and widespread amongst isolates and found on the surface of the bacterium.

Several mechanisms have been employed to identify subunit vaccine candidates, including the use of hyperimmune or post-challenge serum from pigs to identify proteins separated by gel electrophoresis and in silico prediction methods [13–15]. In this report, we utilized a previously reported functional genomic screen to identify subunit vaccine candidates [16]. This screen identifies proteins associated with bacterial fitness and resulted in the selection of RlpB and VacJ as vaccine candidates. The *rlpB* gene (*lptE*) is best studied in *Escherichia coli*. RlpB is a low abundance outer membrane lipoprotein that functions in outer membrane assembly, specifically in mobilizing lipopolysaccharide to the outer membrane’s outer surface, and plays an essential role in cellular viability [17–19]. The *vacJ* gene has been assessed in *G. parasuis* previously [20]. VacJ is an outer membrane lipoprotein that contributes to outer membrane integrity [20]. It has also been associated with stress tolerance, serum resistance, and host cell interaction in *G. parasuis* and other Gram negative pathogens [20–23]. Additionally, the *vacJ* gene was previously assessed for potential as a subunit vaccine against *G. parasuis* in a guinea pig model of disease [15]. In order to assess antigenicity and the potential of recombinant RlpB and VacJ (rRlpB and rVacJ) to stimulate a protective immune response in swine, we vaccinated and boosted naïve pigs with rRlpB and rVacJ 3 weeks apart. Their antibody response was quantified and protection was evaluated through challenge with the *G. parasuis* strain HS069.

## Results

### Comparison of RlpB and VacJ sequence identity

RlpB and VacJ amino acid sequences were compared to evaluate protein sequence diversity among *G. parasuis* isolates. The genome sequence was obtained for 11 *G. parasuis* strains representing 9 different serovars and amino acid sequences of RlpB and VacJ were generated. The *rlpB* gene was obtained for 9 of the 11 strains, the SW114 and 174 genomes are both draft sequences that contain gaps and no *rlpB* was identified. The RlpB amino acid sequence for the remaining 9 strains showed an identity greater than 96% among all strains. A complete *vacJ* gene was present in 9 of the 11 strains. The *vacJ* gene was positioned near the end of a contig in MN-H and was absent from SW140, which may be associated with gaps in the genome of these strains. Amino acid identity among the other 9 strains revealed high conservation, with a 98% or higher identity between isolates.

### Antibody response to vaccination

**Fig. 1:**
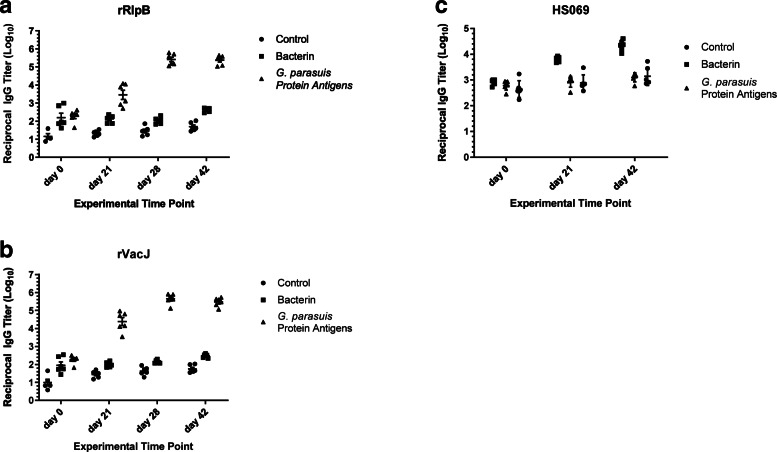
ELISA titers against rRlpB (**a**), rVacJ (**b**), and HS069 (**c**). A significantly higher titer to rRlpB and rVacJ was noted for pigs vaccinated with the *G. parasuis* recombinant proteins than the control animals or the bacterin vaccinated animals. Higher titers to HS069 were seen in HS069 bacterin vaccinated animals. No difference in titer to HS069 was noted between control animals and subunit vaccinated animals

Antibody titers (IgG) were determined by ELISA for rRlpB and rVacJ. Minimal reactivity was seen in animals prior to vaccination. Modest increases in IgG titer to rRlpB and rVacJ were seen in the control and bacterin vaccinated groups prior to challenge, while significant increases in titer with a memory response were seen to both rRlpB and rVacJ for the subunit vaccinated pigs (Fig. 1a and b). Additionally, animals were screened for antibody response to *G. parasuis* HS069. There was an increase in titer for bacterin vaccinated animals, but no change in titer for subunit vaccinated or control animals (Fig. 1c). Titers for bacterin vaccinated animals were significantly higher at day 21 (*p* = 0.03) and day 42 (*p* < 0.01) than that of subunit vaccinated and control animals.

**Fig. 2:**
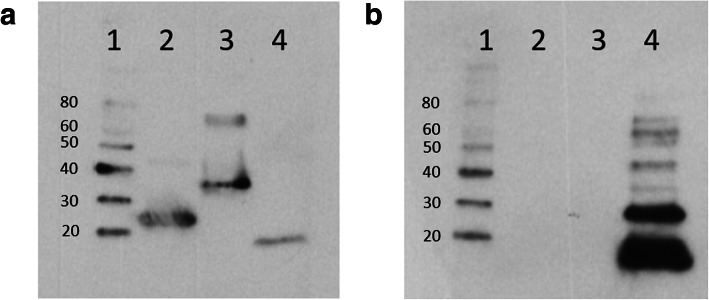
Western blot evaluating antibody specificity. SDS-PAGE of rRlpB (lane 2), rVacJ (lane 3), and *G. parasuis* HS069 sonicate (lane 4) transferred to a PVDF membrane and probed with sera from rRlpB and rVacJ vaccinated pigs (**a**) or bacterin vaccinated pigs (**b**). No reactivity was noted to proteins sized that of RlpB (approximately 25 kDa) or VacJ (approximately 35 kDa) in *H. parasuis* HS069 sonicate when probed with pooled sera from the subunit vaccinated animals. Additionally, no reactivity was noted to rRlpB or rVacJ when probed with pooled sera from bacterin vaccinated animals

Western blotting was utilized to evaluate the specificity of the antibody response. Reactivity to *G. parasuis* HS069 whole cell sonicate was not seen at 25 kDa or 35 kDa, which would correlate to intact RlpB and VacJ respectively (Fig. 2a); however, some reactivity was noted at lower molecular weights. Probing with serum from the bacterin vaccinated animals revealed no reactivity to the recombinant proteins (Fig. 2b).

### Cell mediated immune response

**Fig. 3:**
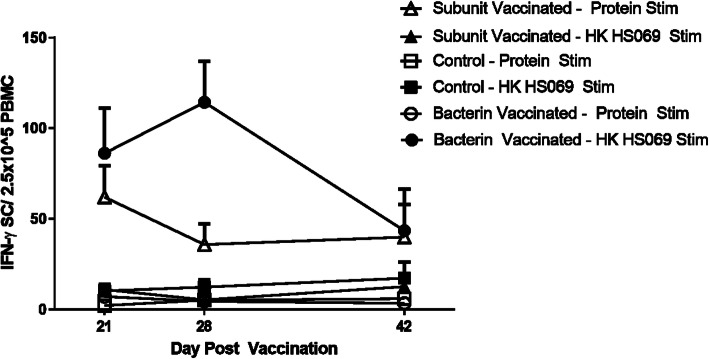
IFN-γ secreting cells responding to stimulation with heat killed *G. parasuis* HS069 and pooled rRlpB and rVacJ. More IFN-γ secreting cells responsive to pooled rRlpB and rVacJ were seen in the subunit vaccinated animals than the control animals (*p* = 0.014) and bacterin vaccinated animals (*p* = 0.006) on day 21 (open shapes). Differences did not meet the statistical threshold on day 28 or 42. Minimal IFN-γ secreting cells were seen in the subunit vaccinated or the control animals in response to stimulation with heat killed *G. parasuis* HS069 (filled shapes). Bacterin vaccinated animals had significantly more IFN-γ secreting cells than subunit vaccinated or control animals at day 21 and 28 (*p* < 0.01)

Peripheral blood mononuclear cells (PBMCs) were collected at the time of boost (day 21), 1 week after boost (day 28), and at challenge (day 42) to evaluate the prevalence of interferon-γ (IFN-γ) secreting cells. Animals immunized with the subunit vaccine were found to have more IFN-γ producing cells showing reactivity to pooled rRlpB and rVacJ than control animals and bacterin vaccinated animals on day 21 (*p* = 0.014 and 0.006, respectively), but differences did not reach the statistical threshold on day 28 and 42 (Fig. 3). Additionally, more IFN-γ producing PBMCs were noted in the subunit vaccine group on day 21 than day 28 or 42, although this was not statistically significant. Minimal reactivity was seen in both the control animals and subunit vaccinated animals to stimulation with heat killed *G. parasuis* HS069, significantly less than that seen in HS069 bacterin vaccinated animals on day 21 and 28 (*p* < 0.01) (Fig. 3).

### Evaluation of protection from *G. parasuis* challenge

**Fig. 4:**
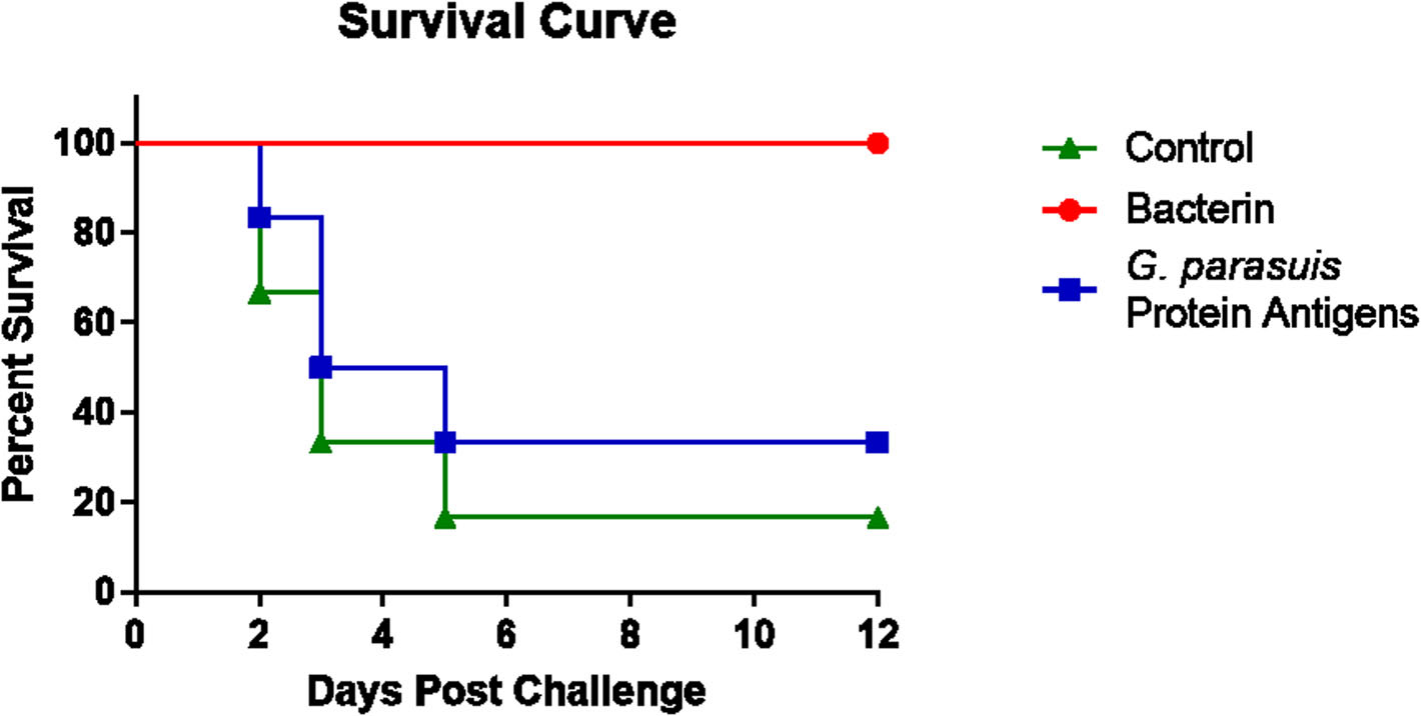
Survival of animals post challenge. Bacterin vaccinated pigs showed significantly better survival post-homologous challenge than the control animals or the *G. parasuis* protein antigen vaccinated animals. No difference was noted between the control pigs and *G. parasuis* protein antigen vaccinated pigs in survival post-challenge

Challenge with *G. parasuis* HS069 caused severe clinical signs (neurologic signs or severe depression) which were exhibited by 5/6 control pigs leading to euthanasia on days 2–5 after challenge (Fig. 4). *G. parasuis* was isolated from at least one site [serosal surface, joint fluid, cerebrospinal fluid (CSF), bronchoalveolar lavage fluid (BALF)] in all five clinically ill control animals. One control pig survived until the end of the study period (day 12 post-challenge) and *G. parasuis* was not isolated from any site samples collected at the time of necropsy.

Four of the six subunit vaccinated pigs also exhibited severe clinical signs and were euthanized on days 2–5 after challenge (Fig. 4). *G. parasuis* was isolated from at least one site in all four of the clinically ill animals. Two subunit vaccinated pigs exhibited no clinical signs during the study period and were culture negative for *G. parasuis* from site samples at necropsy. No difference in survival time was noted between the control animals and those vaccinated with the subunit vaccine (*P* = 0.53).

Bacterin vaccinated pigs showed significantly better protection than control or subunit vaccinated pigs (*P* < 0.01 and *P* = 0.02, respectively). All six bacterin vaccinated pigs survived until the end of the challenge period and exhibited no clinical signs of *G. parasuis* disease (Fig. 4). At necropsy, *G. parasuis* was isolated in the BALF of 3/6 pigs, but no *G. parasuis* was cultured in the serosal swab, joint fluid, or CSF of any of the bacterin vaccinated animals.

## Discussion

*G. parasuis* vaccine development has suffered from difficulty generating a broadly cross-protective vaccine. Many platforms have been attempted: whole cell bacterins, outer-membrane vesicles, live avirulent strains, and a variety of subunit vaccines [5, 6, 13, 15, 24–27]. While homologous protection with whole cell vaccines is often high, those studies investigating heterologous protection show less efficacy [7–10]. This is thought to be associated with the serovar or strain specific response of capsular polysaccharide and some protein antigens. Protein subunit vaccines have good potential to generate a broadly protective immune response if the antigen is widespread, highly conserved, and exposed on the surface of the bacterium or secreted. In this study, we determined two outer membrane proteins, RlpB and VacJ, as subunit vaccine candidates and assessed the capacity of these proteins to protect against *G. parasuis* disease in pigs.

RlpB and VacJ are outer membrane proteins with important contributions to cellular function, including membrane stability and resistance to stressors such as complement [18, 20, 21]. Here, we found RlpB and VacJ play a role in colonization of an in vitro organ culture (IVOC) of swine respiratory epithelium and are highly conserved in isolates of *G. parasuis* of different serovars, making them good subunit vaccine candidates. Additionally, VacJ has previously been utilized in a guinea pig model of *G. parasuis* disease and found to confer good protection [15]. RlpB and VacJ were expressed as recombinant proteins, purified, and used to vaccinate cesarean derived, colostrum deprived (CDCD) pigs. We found the proteins to be highly immunogenic and capable of stimulating a high antibody titer and an anamnestic antibody response following a second vaccination (Fig. 1). We also detected cellular response to the recombinant proteins through detection of IFN-γ secreting PBMCs (Fig. 3). This response was highest at 21 days post vaccination and declined after boost vaccination, consistent with previous reports of protein specific IFN- γ producing cells [16]. Although response to subunit vaccination was noted in both antibody titer and reactive PBMCs, it was unable to protect vaccinated animals from challenge with the homologous *G. parasuis* strain (Fig. 4).

To better understand the lack of protection seen after subunit vaccination, we evaluated the reactivity of anti-sera generated from subunit vaccination and bacterin vaccination. Western blotting revealed no reactivity between pooled anti-sera from subunit vaccinated pigs and whole cell sonicate at the 25 kDa and 35 kDa sizes consistent with RlpB and VacJ; however, reactivity was seen at low molecular weights, which may indicate protein degradation in the sonicate (Fig. 2a). There was also no reactivity between pooled anti-sera from bacterin vaccinated pigs and rRlpB and rVacJ (Fig. 2b). We suspect the absence of reactivity is associated with limited expression of RlpB and VacJ by *G. parasuis* HS069, which is consistent with previous reports in *E. coli* for RlpB [17], or associated with minimal exposure of these lipoproteins at the bacterial surface. Low expression or minimal exposure of RlpB and VacJ in vivo would also prevent high antibody titers to these proteins from providing protection to challenged pigs. Additionally, the recombinant proteins used for vaccination in this study were isolated under denaturing conditions and refolded, which may have altered the presentation of some antigens.

Although this study included small animal numbers in each group (*n* = 6), group size was sufficient to see differences in protection and immune reactivity between bacterin vaccinated animals and subunit vaccinated animals. Additionally, the animals included in this study were crossbred with an unknown genetic background which may increase within group variation; however, this is consistent with production settings.

## Conclusions

Here, we found vaccination of pigs with rRlpB and rVacJ was not capable of inducing a protective immune response, making rRlpB and rVacJ unsuitable for a subunit vaccine to combat *G. parasuis* disease. Further, this report confirms the importance of testing vaccine efficacy in the natural host species. Rodent models for *G. parasuis* do not adequately represent disease in the natural host, as rodents do not develop the typical signs of *G. parasuis* and challenge requires inoculation via an artificial route, typically intraperitoneal or intravenous. These factors make rodent models a starting point for screening subunit vaccine components for *G. parasuis*, but the translation to swine is not exact and further testing in pigs is required.

## Methods

### Bacterial isolates and growth conditions

Transposon mutagenesis and cloning was done with the *G. parasuis* serovar 5 isolate 29755, a virulent strain isolated from the lung of a pig diagnosed with polyserositis [28, 29]. *G. parasuis* HS069, a virulent serovar 5 isolate from the lungs of a pig with respiratory disease caused by *G. parasuis* [30], was utilized for bacterin production and challenge. *G. parasuis* was grown using brain heart infusion base (BHI) (BD Biosciences, San Jose, CA) supplemented with 10% heat-inactivated horse serum and 0.1 mg/mL nicotinamide adenine dinucleotide (NAD). Bacto agar (BD Biosciences) was added to generate solid media.

*Escherichia coli* strains were grown on Luria-Bertani (LB) agar or in LB broth (Thermo Fisher Scientific, Waltham, MA). For expression, *E. coli* was grown in 2YT broth (Life Technologies, Carlsbad, CA). Transformants were selected using 100 μg/mL kanamycin (Sigma-Aldrich, St. Louis, MO).

### Selection of candidate proteins

Selection of candidate proteins was accomplished through a previously utilized protocol combining functional genomic screening using a TraDIS library generated in *G. parasuis* 29755 and in silico bioinformatics to detect outer membrane proteins important to bacterial fitness in a swine respiratory epithelium in vitro organ culture (IVOC) system [16]. Briefly, genes of interest were defined by a loss of fitness in the IVOC system coupled with a transposon insertion in that gene. These genes were then evaluated for subcellular localization using PSORTb (http://db.psort.org/) and LocateP (http://www.cmbi.ru.nl/locatep-db/cgi-bin/locatepdb.py) databases. Localization utilizing literature mining also enabled detection of surface-associated proteins, specifically proteins known to be cell wall anchored or extracellular (lipid-anchored or secretory). Proteins with transmembrane domains in the middle of the coding sequence were excluded. Coding sequences of genes of interest were compared utilizing 11 *G. parasuis* genomes to determine cross protection potential, as previously described [16]. The screening resulted in the selection of two genes of interest, *rlpB* and *vacJ*, which had not been previously published, patented, or assessed for protection against *G. parasuis* in swine.

### Evaluation of VacJ and RlpB sequence identity

The amino acid sequences of RlpB and VacJ were compared using Geneious 9.0.5 (Biomatters Ltd., Auckland, New Zealand). Sequences were obtained for 11 *G. parasuis* strains representing 9 serovars from National Center for Biotechnology Information (NCBI) for SH0165 (CP001321), Nagasaki (APBT00000000), SW114 (APBU00000000), MN-H (APBV0000000), 12,939 (APBW00000000), 29,755 (ABKM00000000), 84–15,995 (APBX00000000), H465 (APBY00000000), D74 (ABPZ00000000), 174 (APCA00000000), and SW140 (APCB00000000). The *rlpB* and *vacJ* genes were extracted from the genomes, translated, and compared using a multiple sequence alignment.

### Cloning and production of rVacJ and rRlpB

The DNA encoding the lipoproteins *rplB* and *vacJ* without the putative secretion sequence (rplB^16–164aa^ and vacJ^19–248aa^) was amplified from *G*. *parasuis* 29755 with the primer pairs listed in Table 1. The amplified PCR products were cloned into the pET-30Ek/LIC expression vector (Millipore Sigma, Burlington, MA) according to the manufacturers’ protocols. The plasmid constructs were verified by PCR and Sanger sequencing.

**Table 1:** Primers utilized to amplify *vacJ* and *rlpB* sequences

Primer	Sequence (5′-3′)
P1 (VacJ_ForA)	GACGACGACAAGATGTGTACTGCTTCTATTGATCCTGAA
P2 (VacJ_RevA)	GAGGAGAAGCCCGGTCAATCAATATTTTTTAGCTGTTCTTC
P3 (RlpB_ForA)	GACGACGACAAGATGTGCGGTTGGCATTTTAAAAA
P4 (RlpB_RevA)	GAGGAGAAGCCCGGTTATTTCGTATTTGCTAACTCTTTTTG

The constructed expression vectors were transformed to *E*. *coli* BL21 (DE3) (Invitrogen, Carlsbad, CA) for expression. Fresh 2YT media (1–6 L) was inoculated with overnight growth of *E. coli* BL21 (DE3) and grown to an OD_595_ of 0.6. Expression was induced with 1 mM IPTG (Sigma-Aldrich). Protein expression was verified with SDS-PAGE from whole cell lysates.

### Proteins purification from batch cultures

#### Isolation and Ni-NTA of rRlpB

Cell pellets containing rRlpB were resuspended in binding buffer (10 mM imidazole, 300 mM NaCl, 50 mM phosphate, pH 8.0) and sonicated on ice in the presence of benzonase and r-lysozyme. Lysates were centrifuged and the supernatant containing soluble rlpB was loaded onto a Ni NTA His-bind Superflow (Novagen) column in binding buffer. The column was washed in binding buffer, followed by 20 mM imidazole, 300 mM NaCl, 50 mM phosphate pH 8.0 before rlpB was eluted with 250 mM imidazole, 300 mM NaCl, 50 mM phosphate pH 8.0.

#### Isolation and Ni-NTA of rVacJ

Cell pellets containing rVacJ protein as inclusion bodies were re-suspended in BugBuster Protein Extraction Reagent (Millipore Sigma, Burlington, MA) in the presence of benzonase and r-Lysozyme. After centrifugation the pellet was re-suspended in BugBuster reagent and pelleted followed by four washes in dilute BugBuster reagent (1:10). The rVacJ containing pellet was dissolved in binding buffer (8 M urea, 0.1 M sodium phosphate buffer, 0.01 M Tris, pH 8.0) and loaded onto Ni NTA His-bind Superflow column (Novagen). The column was washed in binding buffer, then 8 M urea in 0.1 M sodium phosphate buffer, 0.01 M Tris, pH 6.5 before elution in 8 M urea in 0.1 M sodium phosphate buffer, 0.01 M Tris pH 4.5.

#### Anion exchange of rRlpB and rVacJ under denaturing conditions using 8 M urea and dithiotreitol (DTT)

Both the rRlpB and rVacJ containing fractions from the Ni-NTA columns were identified using A280nm and SDS-PAGE and then dialysed into 30 mM Tris/HCl pH 8.5. Most of the rVacJ precipitated due to the removal of the urea, while the rRlpB remained in solution. Urea, DTT and further Tris were added to the dialysate to produce a solution of protein in 8 M Urea, 15 mM DTT, 30 mM Tris pH 8.5, which was incubated for 3 h at 37 °C. The pH of the mixture was adjusted to pH 8.0 and 0.2 μM filtered immediately before loading it onto Source Q - FPLC. Anion exchange was performed using a 25-column volume gradient (low salt buffer: 30 mM Tris pH 8.0, 8 M urea, 2 mM DTT and High salt buffer: 30 mM Tris pH 8.0, 8 M urea, 2 mM DTT, 0.5 M sodium chloride). Eluted peak samples were detected using A280nm and analysed using SDS-PAGE. Proteins were dialysed 3 times in PBS. Overnight dialysis was followed by dialysis in fresh PBS the next day, followed by another change of PBS and a second overnight dialysis. Samples were 0.2 μM filtered to remove any precipitated protein. Purified proteins were submitted to the Iowa State University Protein Facility in Ames, IA for verification by LC-MS/MS.

### Western blotting

Western blotting was used to detect reactivity of sera from bacterin vaccinated pigs to rRlpB and rVacJ and reactivity of sera from subunit vaccinated pigs to whole cell sonicate from *G. parasuis* HS069. The recombinant proteins (2 μg) and *G. parasuis* HS069 sonicate (10 μg) were run on an SDS-PAGE gel and transferred to a PVDF membrane using the iBlot system (Invitrogen, Carlsbad, CA). Membranes were blocked with 10% milk in 1x Tris buffered saline with 0.05% Tween (TTBS) and probed with 5% milk in TTBS containing 1:100 or 1:1000 pooled serum from bacterin vaccinated or subunit vaccinated pigs, respectively. Goat anti-swine IgG conjugated with horse radish peroxidase (KPL, Gaithersburg, MD) at a 1:20,000 dilution in 5% milk in TTBS was used for antibody detection with the Pierce ECL Plus Western Blotting Substrate (Thermo Fisher Scientific, Waltham, MA).

### Vaccination and challenge

All animal work was approved by the USDA-ARS National Animal Disease Center Institutional Animal Care and Use Committee. Eighteen healthy, four-week old cross-bred CDCD piglets were obtained from Struve Labs International (Manning, IA). Pigs were fed a standard finishing diet and kept under laboratory biosafety level II agriculture (BSL2-Ag) conditions at the National Animal Disease Center. After a one-week acclimation, piglets were randomly divided into three treatment groups with six pigs each: vaccinated with adjuvant only (control), vaccinated with *G. parasuis* HS069 bacterin (bacterin), and vaccinated with rRlpB and rVacJ (*G. parasuis* subunit). Control pigs were vaccinated intramuscularly with 2 mL of a 20% Emulsigen D solution. Bacterin vaccinated pigs were inoculated intramuscularly with 2 mL containing 1 × 10^9^ colony forming units (CFU) of formalin inactivated *G. parasuis* HS069 in a 20% Emulsigen D solution (MVP laboratories, Omaha, NE). *G. parasuis* subunit vaccinated pigs were inoculated intramuscularly with 2 mL containing 100 μg each of rRlpB and rVacJ in a 20% Emulsigen D solution. Vaccinations were given twice (day 0 and 21) and pigs were challenged intranasally with 2 mL of 7 × 10^8^ CFU/mL *G. parasuis* HS069 (1 mL per nostril) on day 42 of the study. Animals were monitored post-challenge for signs of systemic *G. parasuis* disease including lameness, lethargy, and neurologic signs. When systemic disease was severe, animals were humanely euthanized. All surviving animals were euthanized 12 days post-challenge. Animals were euthanized by the intravenous administration of an overdose of sodium pentobarbital.

Blood samples were taken on day 0, 21, 28, and 42 in BD Vacutainer serum separator tubes (SST) (Becton Dickinson, Franklin Lakes, NJ). Serum was collected and frozen at − 80 °C until ELISAs were run. Additionally, blood was collected in BD Vacutainer cell preparation tubes (CPT) (Becton Dickinson) with sodium citrate for the isolation of PBMCs on day 21, 28, and 42. At necropsy, samples were collected and culture was performed on the following samples: nasal wash, serosal swab (pleural, pericardial, and peritoneal surfaces), joint fluid from hock or other affected joint, CSF, BALF, and serum.

### Antibody titer analysis via ELISA

Antibody titers to rRlpB and rVacJ were determined using an indirect ELISA. Immulon-2 plates were coated overnight at room temperature with 100 μL of recombinant protein or HS069 in 100 mM carbonate-bicarbonate buffer (pH 9.6). Recombinant proteins were used at the following concentrations: rRlpB at 0.25 μg/mL and rVacJ at 0.125 μg/mL. HS069 was used to coat at a concentration of 0.5 μg protein per mL. Plates were washed three times prior to use with 1X PBS with 0.05% Tween 20 (PBST) and blocked for 2 h with 200 μL of 2% bovine serum albumin (BSA) in PBST. Plates were again washed three times with PBST and probed in duplicate with 100 μL of serial two-fold dilutions of swine antisera in 1% BSA in PBST for 2 h. Following three washes with PBST, protein specific IgG was detected using 100 μL of a 1:25,000 dilution of goat anti-swine IgG conjugated with horse-radish peroxidase (KPL) in 1% BSA in PBST. Plates were incubated for 1 h, washed three times with PBST, and 100 μL of tetramethylbenzidine (TMB) substrate (Life Technologies, Carlsbad, CA) was added to each well. Plates were incubated in the dark for 5 min and stopped with 50 μL of 2 N H_2_SO_4_. The optical density at 450 nm (OD_450_) was measured with a correction at 655 nm (OD_655_). The resulting data was modeled using GraphPad Prism (GraphPad Software, La Jolla, CA) as a nonlinear function of the log_10_ dilution and the log (agonist)-versus-response variable slope four-parameter logistic model. Endpoints were interpolated by using two times the average OD of the gnotobiotic pig serum sample as the cutoff.

### Evaluation of the cell-mediated immune response

The induction of cell-mediated immunity was evaluated following vaccination by enzyme-linked immunosorbent spot (ELISpot) assays. IFN-γ-secreting cells were enumerated after in vitro stimulation with rRlpB and rVacJ. Blood collected in CPT was used to isolate PBMCs as previously described [31]. IFN-γ ELISpot plates were seeded with 2.5 × 10^5^ PBMCs per well with duplicate wells for each treatment. PBMCs were stimulated with the recombinant proteins in a total volume of 0.25 mL (0.5 μg/mL of each individual protein per well). Negative and positive control wells were treated with medium alone or pokeweed mitogen (0.5 μg/mL), respectively. Approximately 18 h after stimulation, ELISpot assays were completed following manufacturer’s recommendations (R&D Systems, Minneapolis, MN). Spots corresponding to IFN-γ-secreting cells were enumerated using an S5UV ImmunoSpot instrument and software (Cellular Technology Ltd., Shaker Heights, OH). The number of IFN-γ-secreting cells was calculated for each treatment in each group using the average of the duplicate wells for each pig.

### Statistical analysis

Statistical analysis was completed using GraphPad Prism 7. Survival curves were generated by the product limit method of Kaplan and Meier and compared using the log-rank test. Log_10_ antibody titers were compared using two-tailed Student’s *t* tests to evaluate differences between groups. Cell mediated response was analyzed using a two-way analysis of variance (ANOVA) with Tukey’s multiple comparison posttest. Statistical significance was designated at *P* < 0.05.

## Data Availability

Not applicable.

## Abbreviations

BALF: Bronchoalveolar lavage fluid
BHI: Brain heart infusion
BSA: Bovine serum albumin
CDCD: Cesarean derived, colostrum deprived
CFU: Colony forming units
CHAP: 3-[(3-cholamidopropyl)-dimethylammonio]-1-propanesulfonate
CPT: BD Vacutainer cell preparation tubes
CSF: Cerebrospinal fluid
ELISpot: Enzyme-linked immunosorbent spot
IFN-γ: Interferon gamma
IgG: Immunoglobulin G
IVOC: In vitro organ culture
LB: Luria-Bertani
LC-MS/MS: Liquid chromatography with tandem mass spectrometry
NAD: Nicotinamide adenine dinucleotide
PBMC: Peripheral blood mononuclear cell
PBST: 1X PBS with 0.05% Tween 20
PVDF: Polyvinylidene fluoride
rRlpB: Recombinant RlpB
rVacJ: Recombinant VacJ
SDS-PAGE: Sodium dodecyl sulfate polyacrylamide gel electrophoresis
SST: BD Vacutainer serum separator tubes
TMB: Tetramethylbenzidine
TTBS: 1X Tris buffered saline with 0.05% Tween
